# Consistencies and contradictions in different polymer models of chromatin architecture

**DOI:** 10.1016/j.csbj.2023.01.033

**Published:** 2023-01-24

**Authors:** Amanda Souza Câmara, Martin Mascher

**Affiliations:** Leibniz Institute of Plant Genetics and Crop Plant Research (IPK) Gatersleben, 06466 Stadt Seeland, Germany

**Keywords:** Chromatin architecture, Chromatin dynamics, Copolymer models, Mechanistic models, Polymer physics

## Abstract

Genetic information is stored in very long DNA molecules, which are folded to form chromatin, a similarly long polymer fibre that is ultimately organised into chromosomes. The organisation of chromatin is fundamental to many cellular functions, from the expression of the genetic information to cell division. As a long polymer, chromatin is very flexible and may adopt a myriad of shapes. Globally, the polymer physics governing chromatin dynamics is very well understood. But chromatin is not uniform and regions of it, with chemical modifications and bound effectors, form domains and compartments through mechanisms not yet clear. Polymer models have been successfully used to investigate these mechanisms to explain cytological observations and build hypothesis for experimental validation. Many different approaches to conceptualise chromatin in polymer models can be envisioned and each reflects different aspects. Here, we compare recent approaches that aim at reproducing prominent features of interphase chromatin organisation: the compartmentalisation into eu- and heterochromatin compartments, the formation of a nucleolus, chromatin loops and the rosette and Rabl conformations of interphase chromosomes. We highlight commonalities and contradictions that point to a modulation of the mechanisms involved to fine degree. Consolidating models will require the inclusion of yet hidden or neglected parameters.

## 1 Introduction

Chromosomes are the top-level organisational unit of hereditary information. As such, their physical arrangement in the nucleus must not impede, and may possibly assist in, the expression of that information [1], [2]. A very prominent architectural feature is the visible distinction of eu- and heterochromatin, evident from chromosome staining pattern. Less dense and easily accessible to other proteins, euchromatin is enriched in genes; heterochromatin is denser and consists mostly of non-transcribed elements. Both chromatin domains are associated with specific histone modifications and a nuclear localisation that governs gene expression. But the mechanisms that drive this and other architectural features of chromatin organisation are not well understood.

Misteli suggested that there are five basic organising principles [3]: 1) self-interactions mediated by chromatin binding proteins; 2) interactions with rapidly binding proteins in response to the cellular environment 3) phase separation into domains, like eu- and heterochromatin, that preferentially interact with themselves rather than with others; 4) constrictions due to architectural elements like the nuclear envelope; and 5) the stochastic motion of chromatin that gives rise to heterogeneity between cells. The modulation of these mechanisms creates a range of conformations, each with a certain probability of showing a pattern of gene expression. Marenduzzo et al. suggested that entropy (a measure of disorder; the second law of thermodynamics posits that total entropy of a system increases during spontaneous process) is also a major mechanism effecting phase separation and driving the formation of protein clusters and chromatin loops [4].

Chromatin behaves as a long polymer [5]. For example, it diffuses slowly (anomalous diffusion exponent < 1), it rarely reptates (a motion like the entanglement of snakes in the nest), and it self-interacts by forming loops. Polymer simulation probes physical properties of chromatin regulation, beyond the genome sequence – like the mechanisms listed above. It builds comprehensive models to support experimental results from varied techniques - chromosome conformation capture sequencing technology (Hi-C) [6], super-resolution microscopy and mechanical manipulation. Recent technological developments in these fields have furthered our understanding of chromatin organisation.

In addition to the polymeric features of eukaryotic chromosomes, there must be additional physical changes to chromatin polymers to modulate the organisation and accommodate the diverse processes of life. In this review, we discuss some recent literature on modelling chromatin as a polymer with different local properties. Conceptually diverse approaches succeeded in recreating some of the major characteristics of chromatin architectures in model plants and animals such as eu- and heterochromatin compartments, the nucleolus, chromatin loops and the rosette and Rabl conformations. As computational power and available genomic data grow rapidly, polymer simulations are becoming more sophisticated and comprehensive. However, diverse evolving approaches to model the chromatin polymer sometimes reach contradictory conclusions. Here we compare these approaches to better understand the biological mechanisms that they simulate.

## 2 A focus on mechanistic copolymer models of chromatin

There are two main polymer simulation approaches: mechanistic approaches, also called forward or first-principles approaches, and data-driven or inverse approaches [7]. Forward approaches start with assumptions on the mechanisms of chromosome dynamics, build models based on the physical properties of polymers, and rely on few experimental observations (for instance from Hi-C and microscopy). Inverse approaches, too, consider the physical properties of polymers and experimental observations, but are trained on larger data sets from different experiments, like Hi-C, chromatin immunoprecipitation sequencing (ChIP-seq), RNA sequencing or fluorescence in situ hybridisation, to make predictions on new data sets [8]. While inverse approaches aim to place sequence elements in a spatial context that reproduces experimental observations, forward approaches aim to reproduce the dynamics of chromatin inside a specific spatial context [9]. This review focuses on forward approaches, which can teach us about possible mechanisms that result in different chromosome conformations.

Most models describe chromatin as a beads-on-a-string polymer – a single chain, with no bifurcations, where each bead, or monomer, represents one or more nucleosomes or a chromatin portion (Fig. 1A). How much chromatin is represented by each portion depends on the desired level of details and the available computational resources. Two or three physical forces describing the interactions of bonded and non-bonded monomers are enough to reproduce the general chromatin motion [10]. These forces may define covalent bonds, bending, attraction and repulsion and are tuned according to properties of the polymer fibre, like stiffness and diffusion rate.

**Figure:**
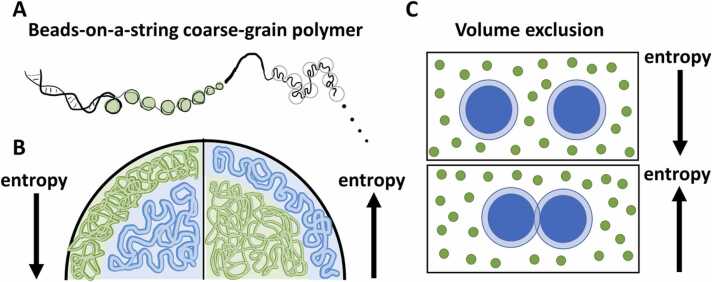
Fig. 1 Beads-on-a-string model, volume exclusion and system entropy. A) Scheme of a beads-on-a-string coarse-grain polymer model of the chromatin fibre. Each bead (black empty circles) represents a portion of the chromatin fibre, which can be one or more nucleosomes. B) Thick (blue) and thin (green) polymers occupying peripheral or central positions inside a sphere. Both polymers lose possible conformations and entropy when squashed against the borders of the sphere, but the loss is bigger for the thinner and more flexible polymer [13]. C) A mixture of big and small particles. The excluded volume of the big particles is showed in light blue. It is the volume around each particle that is inaccessible to the others. When big particles come together, the sum of their excluded volumes decreases and more space is available for the smaller particles, increasing the entropy of the system ([16]).

Polymers whose monomers have different characteristics are called copolymers, or heteropolymers. Copolymer models consider different local properties of the chromatin fibre. For example, nucleosomes with different histone marks, associated with eu- or heterochromatin, can be modelled as monomers with different stiffness or interaction forces. Models with different stiffness or other properties of the chromatin fibre (thickness or compaction) emphasise the entropic effects on the dynamics of the nucleus. Models with specific interaction forces emphasise the roles of attraction between the different nuclear elements (lamina or nucleoli) to the nuclear organisation.

In the following, we describe some mechanistic copolymer models to illustrate how different architectures can be investigated. These examples reproduce, to some extent, heterochromatin domains, the nucleolus, chromatin loops, and the traces of mitotic condensation in interphase.

## 3 Heterochromatin domains clustering and positioning

Heterochromatin was first observed by Heitz [11] and defined as chromosomal regions that remained compact throughout the cell cycle. Nowadays, it is known to be gene-poor, transcriptionally inactive, and associated with specific histone marks and chromatin-binding proteins [12].

Cook and Marenduzzo designed a very elegant set of simulations to assess how entropy affects the organisation of polymer chains with different properties – length, stiffness, thickness, compaction and arrangement – mimicking the differences between eu- and heterochromatin [13]. Sets of conformations were analysed according to the organisational trend of each type of polymer relative to an enclosing spherical compartment, which represented the nuclear envelope.

Stiff, thick and compact chains tended to occupy the borders of the nucleus. This is in accordance with many cytological observations stating that heterochromatin is mainly found at the nuclear periphery [14], and it can be explained in terms of the entropy of the system. When squashed against the borders, flexible chains loose more possible configurations (entropy) than rigid chains [13]. Thus, rigid chains accumulate at the borders to free space for flexible chains at the centre, increasing the entropy of the system (Fig. 1B).

The computational study by Oh suggested another factor that increases the entropy of the system [15]. The authors simulated small groups of nucleosomes that were spatially restrained to the group’s centre, looking like clutches of eggs in the nest. Changing the restraints to low or high density formed eu- and heterochromatin clutches, respectively. They observed that pairs of heterochromatin clutches attract each other upon crowding of surrounding molecules, whereas pairs of euchromatin clutches do not. Heterochromatin clutches behave as hard spheres (euchromatin would form softer spheres) in a crowded solution [16]. The entropy of the solution increases as hard spheres fit into ever smaller volumes – a phenomenon known as volume exclusion or depletion attraction (Fig. 1C). In addition, crowding in the nucleus was seen in experiments with live cells by Bancaud et al. [17]. These authors report different molecular diffusion rates for proteins inside euchromatin, heterochromatin and nucleoli, which they suggest can be attributed to volume exclusion, diffusion hindrance and enhanced affinity inside dense nuclear compartments, all of which are promoted by crowding. However, this mechanism relies on the pre-existence of dense structures, like hard spheres or heterochromatin clutches, that attract each other and form a larger dense structure, like a heterochromatin compartment.

The above-mentioned simulations by Cook and Marenduzzo also start with models of eu- and heterochromatin physically different a priori in stiffness and thickness [13]. The biological origin of such differences is chemical modifications of chromatin like methylation or molecular effectors like the attachment of protein or protein complexes to chromatin. One such protein is Heterochromatin Protein 1 (HP1). Highly conserved in animals, and with functional analogues in plants [18], it has an intrinsically disordered region that causes liquid-liquid separation in vitro and in vivo. Strom et al. investigated the link between dynamics of heterochromatic domains and the liquid separation engendered by HP1 [19]. They concluded that phase separation is key to the formation of distinct multichromosomal domains and underlies their association with, or disassociation from, genomic regions. H2B.8, a histone variant with an intrinsically disordered region at the tail was observed to aggregate transcriptionally inactive AT-rich euchromatin regions through phase separation [20]. It is present in sperm cells of flowering plants, which lack protamine (a protein that promotes chromatin condensation of animal sperm cells), and helps to condense euchromatin, besides the usually condensed heterochromatin, and to decrease the size of sperm nuclei. This example suggests that different proteins may be recruited to form even more than two separate phases of chromatin.

**Fig. 2:**
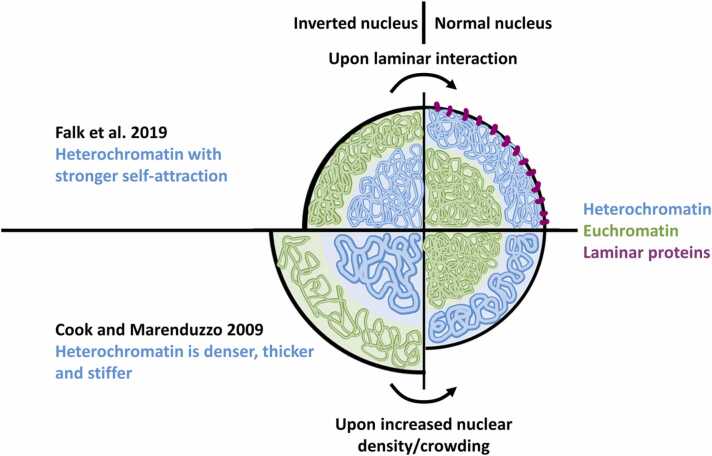
Two proposed mechanisms to change between normal and inverted nuclei. The sphere represents the nucleus membrane, and each quartile represents one model in the normal (right) or inverted (left) conformation. Upper quartiles represent the mechanism modelled by Falk et al., which emphasises the role of specific interaction forces [22]. On the left, heterochromatin (blue) appears in the centre because of its self-attraction, stronger than of euchromatin (green). But heterochromatin moves to the periphery (right) upon attraction to the nuclear membrane mediated by laminar proteins (purple). Bottom quartiles represent the mechanism modelled by Cook and Marenduzzo, which emphasises the role of entropy [13]. Heterochromatin is denser, thicker and stiffer than euchromatin and appears at the centre of the nucleus sphere. But it moves to the periphery when the nuclear volume decreases and the nuclear environment becomes more crowded and dense.

Phase separation is consistently described to be the mechanism separating eu- and heterochromatin, but it fails to explain the positioning of heterochromatin, which usually sits at the borders of the nucleus. Rod cells of nocturnal animals are the perfect example to study the positioning of heterochromatin, due to their inverted nuclei. The unusual central position of a single heterochromatin domain reduces the scattering of the light that passes through it, enhancing the nocturnal vision of these animals [21]. Recently, Falk et al. simulated inverted nuclei with copolymer models [22]. They modelled eu- and heterochromatin monomers (each representing ∼ 200 nucleosomes) by changing the strength of the interactions between them instead of varying physical properties of the polymer chains, like thickness and stiffness (as did Cook and Marenduzzo [13]). They found that stronger self-interaction makes heterochromatin monomers form one single large multichromosomal domain at the centre of the nucleus. That configuration is mediated by phase separation and recreates inverted nuclei. Only when attraction to the nuclear envelope is considered does heterochromatin organise itself into smaller compartments at the border of the nucleus, which reflects the conventional configuration (Fig. 2).

Multi-phase-field mathematical models agree that the attraction to the nuclear envelope is essential for heterochromatin to accumulate at the nuclear borders [23]. But these models and Falk et al.’s models fail to account for experimentally observed higher densities of heterochromatin, which Cook and Marenduzzo et al. and others suggest are essential to put heterochromatin at the border of the nucleus (Fig. 2) [24], [25], [26]. The copolymer model of Jerabek and Heermann simulates heterochromatin with higher densities than euchromatin and places heterochromatin at the centre of the chromosomal territories, but possibly because nuclear confinement was not simulated [27]. Simulations with stiff heterochromatin by Cook and Marenduzzo and Awazu reproduced inverted nuclei only upon increased nuclear size [13], [24]. But inversion of the nuclear configuration is hard to explain solely by the entropy of different nuclear sizes because rod cells exit mitosis with conventional nuclei, which invert as the cells mature and the nuclei get smaller (not bigger, as suggested by Cook and Marenduzzo and Awazu). Moreover, in mice, the inverted nuclei of rod cells are just as small as other nuclei with conventional configuration, like the ones of spleen lymphocytes [21].

Taken together, a consensus holds that intrinsic compaction of heterochromatin maintains its clustering into distinct domains mediated by phase separation. The positioning of heterochromatin has been ascribed to each of a set of different effectors, like crowding and laminar interactions, but it is more likely to be a combination of them. In the future, a link between these two as yet unconnected facts may be established by simulating chromatin fibres with different attraction patterns as well as different topologies to find factors that determine how heterochromatin clusters and positions itself.

## 4 The nucleolus in *Arabidopsis thaliana*

*Arabidopsis thaliana* (henceforth Arabidopsis) is a model plant. As such, the species has a near-complete reference sequence assembly of its exceptionally small genome (∼ 120 Mb). Comprehensive genomic data have been collected, and cytologists have studied the structure and organisation of the Arabidopsis genome in much greater detail than in any other plant species. Because of its small genome size and few chromosomes (2n = 2x = 10), Arabidopsis is ideally suited to whole genome simulations. Its nuclear architecture is well described [28], with easy-to-identify dense heterochromatic chromocenters at the nuclear periphery and a big central nucleolus, containing the ribosomal DNA (which is spatially clustered in nucleolus organiser regions (NORs) of the genome) and many proteins associated with ribosomes. Taking into account the available data and the prior knowledge about nuclear architecture, two mechanistic models were proposed for the Arabidopsis genome interphase organisation, which we discuss below.

**Fig. 3:**
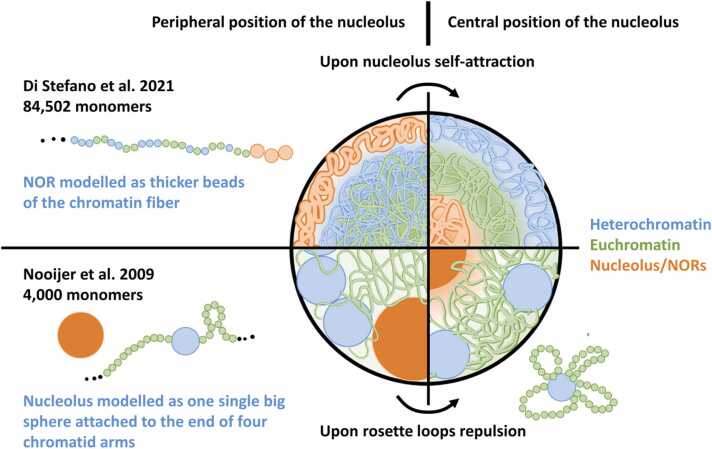
Two proposed mechanisms to change the nucleolus position from the nuclear periphery to the centre. The sphere represents the nucleus membrane, and each quartile represents one model of the nucleolus in the nuclear periphery (left) or in the centre (right). Upper quartiles represent the mechanism modelled by Di Stefano et al. [29], in which heterochromatin has strong repulsion against other chromatin domains and the nucleolus is modelled as thick beads of the chromatin fibre. The nucleolus localises at the periphery (left) and moves to the centre (right) only upon self-attraction of its beads. They emphasise the role of specific interaction forces. Bottom quartiles represent the mechanism modelled by Nooijer et al. [30], in which heterochromatin is modelled as big spheres, one for each chromatid representing the centromeres and the nucleolus is a single sphere, bigger than centromeres, attached to four chromatid arms. When the chromatid arms are modelled as linear polymers, possibly with loops, centromeres and the nucleolus localise at the nucleus centre. When the chromosomes are modelled in the rosette configuration, centromeres remain peripherical but apart from each other and from the nucleolus, which moves to the centre of the nucleus. They emphasise the role of entropy.

Di Stefano et al. put forward a copolymer model for Arabidopsis chromosomes with different topologies and interactions [29]. Specific interactions were added for eu- and heterochromatin monomers guided by the epigenetics landscape as inferred by ChIP-seq data for histone modifications – specifically, H3K4me2 and H3K4me3 (related to euchromatin), H3K27me3 (related to facultative heterochromatin) and H3K9me2 (related to constitutive heterochromatin). Chromatin was spherically confined to mimic the nuclear environment. A total of 84,502 monomers, 3 kb in size and 30 nm in diameter, made up most of the genome. But monomers at NOR and telomeres were simulated with a 132 nm diameter (Fig. 3). Thicker monomers were positioned initially at the nuclear periphery. But as self-attraction was introduced in the model, these monomers clustered into a single big sphere at the centre of the nucleus, like the nucleolus. The presence of the nucleolus at the centre forced the heterochromatin to the periphery. The positioning of the nucleolus and repulsion forces between monomers of heterochromatin were the major drivers of global chromatin organisation in the simulated models, which accurately reproduced the patterns in Hi-C contact matrices of the Arabidopsis genome.

Nooijer et al. simulated the genome of Arabidopsis with a total of 4000 monomers of 169 nm diameter [30]. Centromeres were modelled as one big monomer (1900 nm diameter) at the centre of the chains, and the entire nucleolus as one even bigger monomer (3000 nm diameter) attached to the end of four chromatid arms (as only chromosomes 2 and 4 have NORs). They tested three different chromosome architectures: 1) linear chains, 2) linear chains with one big loop in each arm, and 3) rosette-like chains with three to five loops emanating from the centromere (Fig. 3). In all architectures, except for the rosette-like model, centromeres and nucleoli clustered at the nuclear periphery. The rosette loops prevented centromeres of distinct chromosomes from clustering, and centromeres stayed put at the periphery as the nucleolus moved to the centre of the nucleus. This positioning of nucleolus and centromeres reproduced microscopic observations. The one thing it failed to reproduce was the clustering of centromeres into chromocenters at the periphery while the nucleolus sits at the centre.

In the models of Di Stefano and Nooijer, thicker monomers accumulate at the nuclear periphery without any specific forces in the model mandating that. Possibly, volume exclusion enhances the entropy of smaller monomers at the interior of the nucleus as observed in the simulations of Cook and Marenduzzo [29]. Both models also concur in the repositioning of the nucleolus to the centre but explain it by different mechanisms. According to Nooijer et al., loops emerging from the centromeres repeal other thick monomers and especially the nucleolus, which, because of the repulsion of rosette loops, moves away from the peripherally located centromeres to the nuclear centre. Di Stefano et al. attributed that configuration solely to self-attraction of the NOR-forming monomers. Consistently, strong self-attraction also puts constitutive heterochromatin at the centre of inverted nuclei in the model of Falk et al.

The nucleolus is a common structure in eukaryotes. It presents general features and mechanisms, which can be studied across different organisms. Particularly, the separation of the nucleolus from the rest of the chromatin as a nucleolar body has been largely understood as phase separation of liquid droplets. Like droplets, the nucleolus has a round shape and the dynamics of a fluid insofar that it is able to fuse smaller nucleolar bodies into one big droplet, as simulated by Di Stefano et al. [29]. But more than one nucleolar body can be experimentally observed as a stable state, which Qi and Zhang explained as an effect of the surrounding chromatin network using polymer simulations of the human genome [31]. They modelled nucleolar particles as unconnected beads (mimicking nucleolar proteins) that attract both each other and some regions of the chromatin, the NOR. Chromatin was modelled as a beads-on-a-string copolymer, with 1 Mb resolution, and different interaction strengths between monomers of euchromatin, heterochromatin or centromere were used following a previous model of GM12878 cells based on Hi-C data. They tried different strengths for the nucleolar particles-NOR interactions and concluded that the stronger this interaction, the higher the number of nuclear bodies. This interaction seems to increase chromatin network constraints, decrease the diffusion of nucleolar droplets and heighten entropic barriers between them; possibly like the entropic barriers between centromeres of two distinct chromosomes in rosette shape, as described by Nooijer et al. [30].

The simulations of nucleolar droplets by Qi and Zhang revealed mechanisms described in both Di Stefano et al. and Nooijer at al. models – the attraction of nucleolar particles and NORs, and the entropic repulsion of nuclear bodies due to chromatin’s polymeric nature. But the central place accorded to NORs in their models may not be universal, as NOR may not be as prominent in species with larger genomes and chromosomes. Other models put more weight on specific forces for the repulsion of heterochromatin which could be achieved by thicker heterochromatin monomers subject to phase separation. Further simulations that consider different (relative) sizes and topologies for heterochromatin and the existence of chromatin loops might reconcile the conclusions on the forces driving the positioning of the nucleolus, and shed light on nuclear dynamics in other species.

## 5 Causes and consequences of chromatin loops

Loops are common in long polymers. Interactions network within domains are formed by bonds either between fixed monomers or between random monomers. Most models discussed here present loop-forming interactions, but their effects on the chromatin organisation was poorly described and depend on the mechanism that forms them.

**Fig. 4:**
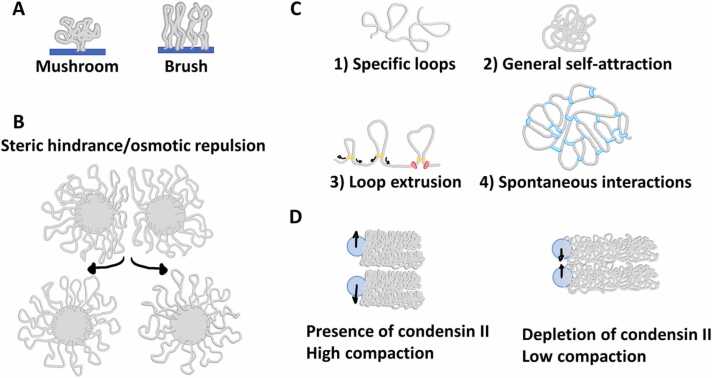
Conformations of chromatin loops and their repulsive effects. A) Low concentration of loops on a surface leads to a mushroom conformation of the loops. At high concentrations and with space restricted, loops adopt a brush conformation. B) Spheres with loops in a brush conformation repel each other due to steric hindrance, or osmotic repulsion, between the loops. C) We identify four possible mechanisms of forming chromatin polymer loops: 1) attraction between fixed monomers (specific loops); 2) a general self-attraction of all monomers; 3) loop extrusion by loop extruders (yellow), forming growing loops at any position, but modulated by the presence of other effectors (red) bound to the chromatin fibre; and 4) binding, mediated by proteins (blue) or intrinsic attraction, of monomers that spontaneously approach each other. D) Scheme of the interaction between centromeres (blue) of chromosomes in the Rabl configuration observed by [52]. When condensin II is present in mitotic chromosomes, the brush conformation of highly compacted chromatid arms repel the centromeres. And when condensin II is absent in mitotic chromosomes, the loose conformation of chromatid arms allow attraction of centromeres.

Polymer loops lack extremities. That prevents reptation and entanglement. When many loops are intertwined at their bases, for example in rosette-like structures or bottle-brush models, steric hindrance appears between the loops. Steric hindrance here means that neighbouring loops obstruct each other’s space, which limits the number of possible conformations that they can adopt. Like polymers grafted to a surface, at low concentration of polymers (or loops) more surrounding space is available and they form a mushroom conformation; at high concentration of polymers (or loops), the surrounding space is restricted, and they adopt a brush conformation (Fig. 4A) [32]. When two rosette-like structures, with loops in the brush conformation, come closer, the concentration of loops between them increases, causing an osmotic repulsion between the two structures (Fig. 4B) [33]. In chromosomes, these properties of loops promote chromosome territories and chromatid segregation [34], [35].

Chromatin loops certainly play an important role in chromosome organisation, but it is hard to assess their effects in simulations when they are not accounted for explicitly. Cook and Marenduzzo studied compaction by loops and by a general stronger self-attraction of monomers in the same chain (Fig. 4C) [13]. Either way, chromosomes occupied the same volume, but self-attracting chromatin, because of its higher rigidity, sits preferentially at the nuclear periphery.

All the models discussed so far propose that distinct chromatin domains have different mechanisms that govern how they compact or interact. Bohn and Heermann proposed a Dynamic Loops model in which regions with high or low levels of gene expression (corresponding to transcriptionally active and repressed chromatin) are both organised into loops, but to different degrees. Distant monomers eventually approach each other by diffusion [36]. When this happens, they form a transient bond (Fig. 4C). The affinity between these two monomers determines how long the bond between them lasts. Inactive regions have higher self-affinity, and bonds and loops therein are more stable. Equilibrium simulations of this model showed that inactive regions were denser, with slower diffusion and with a slower decay in the contact probability – meaning more interactions of linearly distant monomers or more and longer loops. Like the Dynamic Loops model, the Strings and Binders Switch (SBS) model proposed by Nicodemi et al. also reproduced contact probabilities [37], [38]. It does so by simulating non-connected particles that interact with specific monomers of the chromatin polymer as affinities and concentration vary, mimicking chromatin-binding proteins. Both the Dynamic Loops and the SBS models directly promote only local interactions. Yet they indirectly reproduce larger domains of different chromatin states. Such an interconnected organisation could give chromatin gel-like features, which means less fluidity and flexibility to chromatin and surrounding proteins. Mechanical manipulation of a single locus in interphase showed that chromatin is rather a weak gel, with brief interconnections [39].

However, spontaneous 3D interactions, such as the ones considered in the Dynamic Loops and the SBS models, fail, among other things, to distinguish between loci on different chromosomes, and are not sensitive to locus orientation [40]. The loop extrusion model overcomes these difficulties by considering loops that progressively grow along the chromatin fibre [41]. Crucial to this model are loop extruding factors (LEFs), whose real-life equivalent are most probably proteins of the Structural Maintenance of Chromosomes family (SMC) and the complexes formed by them: condensins I and II and cohesin. LEFs bind anywhere and reel over the chromatin fibre to form enlarging loops (Fig. 4C). They are regulated by the presence of other chromatin-bound factors. Such models successfully explained the existence of Topological Associating Domains and the compaction of different metaphase conformations by the presence of boundary elements halting the action of LEFs [42], [43], [44], [45]. Loop extrusion was also observed to lead to optimal brush structures [33], perhaps in similar ways as the growing preparation strategy of grafting polymers from surfaces that leads to higher concentration of grafted polymers [32].

In addition to the different compaction mechanisms listed here – general affinity, spontaneous loops, specific loops and loop extrusion – epigenetic modifications possibly play a role in determining compaction levels, and chromatin associated with them may have preferential relative positions in the nucleus. Future studies could investigate the interplay between well-studied epigenetic modifications such as DNA methylation and histone modifications, and biophysical mechanisms. Most recently, Fujishiro and Sasai considered spontaneous loops and loop extrusion in the same model [46]. They simulated 8–30 polymer chains of 500 monomers each, all subjected to loop extrusion, and each of either eu- or heterochromatin type, differing with regard to the time that the spontaneous interactions last (which is longer for heterochromatin). This strategy resulted in denser heterochromatin chains and a final potential energy function that was mainly repulsive for both types of chains, like Di Stefano et al. found for Arabidopsis [29]. They observed that (i) phase separation again explained the differences in the locations of eu- and heterochromatic domains in these models, and (ii) that heterochromatin localises at the nuclear periphery without explicit interactions with the lamina, which can further stabilise or perturb the system. Fujishiro and Sasai further use the final potential energy function for each type of chromatin region to simulate whole human genomes, at lower resolution, but implicitly accounting for independent interaction mechanisms [46]. This approximation could be a way to increase the complexity of the models without increasing computational cost, and enhance, for example, existing metaphase chromosome models, where heterochromatin condensation blends in with overall condensation via loop extrusion. In metaphase, SMC complexes abound and accumulate in certain places [47], [48]. Attempts to fit metaphase models to Hi-C data have suggested that there might be different arrangements of chromatin loops (as different helical turn lengths) along the chromosome [49]. But the appearance of visible heterochromatins in chromosome banding via staining with, for example, Giemsa or Quinacrine, yet awaits explanation and points to compaction mechanisms that no helical model has so far taken into account.

## 6 “Metaphase memory” and decondensation

We have learned a lot about the compaction of chromosomes in the past decade. Still, mechanistic models for the decompaction process from metaphase to interphase, which might play an important role in interphase chromosome organisation, are tentative at best. Rosa and Everaers calculated that the cell cycle proceeds too rapidly for the 3D conformation of chromosomes to equilibrate because of their low diffusion rates [50]. Hence, the interphase organisation keeps a “memory” from metaphase. In fact, support for this idea goes back to Rabl [51]. Rephrased in modern terms, the “Rabl conformation” refers to the fact that centromeres and telomeres cluster at opposite ends of the cell while short and long chromosome arms juxtapose, as they do when the kinetochore pulls them to opposite poles after metaphase. Fujishiro and Sasai simulated the Rabl conformation in the human genome by pulling linearly condensed chromosomes by their centromere loci [46]. Interphase organisation was then obtained by allowing the chromosomes to expand with repulsive forces between different chromatin regions and without constraints of SMC complexes. Even after this relaxation, the chromosomes still hold the Rabl conformation.

Arabidopsis chromosomes are short, and may, for this reason, reach rapidly an equilibrium conformation in interphase that is not Rabl but rosette-like. Even so, polymer simulations, starting from compacted chromosomes that have just been pulled at the centromeres, better reproduced contact probability patterns from Hi-C at larger distances in Arabidopsis [29].

In another study, Hoencamp et al. modelled chromosomes with different lengthwise compactions [52]. That process may be promoted by chromatin loops effected by condensin II during mitosis. As chromosomes were compacted lengthwise in the model of Hoencamp et al., several hallmarks of interphase chromatin did not emerge: absent were the high concentration of chromatin loops packed in the brush conformation, the loss of interchromosomal interactions (due to osmotic repulsion), and the clustering of centromeres. Less compact chromosomes, by contrast, intermingled and their centromeres clumped together (Fig. 4C), as seen in the model of Nooijer et al. [30], where centromeres are repelled by osmotic repulsion between loops of the rosette conformation. These examples motivate future modelling of the decondensation process and may elucidate the differences between fast-cycling and quiescent cells.

## 7 Summary and outlook

Chromosomes are long polymers and partake in the universal characteristics of such molecules, which can be reproduced by computational models without accounting for DNA sequence [53]. The physics of polymers guarantees that chromatin organisation behaves in a predictable manner. To model the plasticity and heterogenicity of chromatin organisation across species and tissues, we need copolymer models, which account for differences along the chromatin fibre, like DNA methylation, histone modifications and chromatin-binding proteins.

Arguably the most important difference in chromatin states is that between euchromatin and heterochromatin. How does heterochromatin compartmentalise into different locations of the cell nucleus? Some models presented here contradict each other over the role of the nuclear envelope, but each simulates different properties of heterochromatin. It is possible that all the different properties researchers simulated may be present and modulating the organisation of heterochromatin, instead of regulating it as on-off switches. The same may apply to the positional organisation of the nucleolus, which has in its own right a complex architecture with multiple phases [54].

Models such as those of Nooijer et al. and Cook and Marenduzzo explain the general relative distribution of membraneless compartments in the nucleus by forces like entropy and phase separation, which are not specific to living cells [13], [30]. Apart from stiffness and thickness of the chromatin polymer, different repulsion forces between non bonded chromatin monomers, as modelled by Fujishiro and Sasai for eu- and heterochromatin [46], can also reproduce the heterogenous movement and compaction of the chromatin to simulate entropic and phase separation effects. Those models convincingly argue that these effects indeed drive the organisation of chromatin domains, whose topology can be modelled in different ways.

Inverse models considering epigenomic data excel at recapitulating Hi-C contact patterns, but, because of potential overfitting, make conceptual generalisations and mechanistic insights harder. Mechanistic models that consider regions with different biophysical properties, besides their specific interaction, may draw a more complete picture of the genome organisation. For instance, the models by Di Stefano and Falk et al. reproduce phase separation and peripheral positioning of heterochromatin, by modulating the forces of interaction of domains with themselves and with others [22], [29]. But their density and level of compaction are constant and those two models do not concur about the major forces driving heterochromatin organisation – repulsive in one and attractive in the other. Nevertheless, they have achieved much needed conceptual advances that motivate future research. The modulation of interaction forces that they probed is also a common approach to find a balance of different effectors. For example, Qi and Zhang modulated the strength of the interaction between NOR and nucleolar particles. These models demonstrate how to optimise the variety of possible interactions involving chromatin in a way that best reproduce experimental observations.

The polymer models presented vary greatly in the way they represent chromatin loops too. We have still much to find out about the roles loops play in guiding specific and non-specific interactions between epigenetically different regions. Loop sizes are still below the resolution level of whole genome models, but proxies such as potential energies that implicitly account for loops can be used as did Fujishiro and Sasai [46]. Future polymer models should aim for a comprehensive list of possible mechanisms driving chromosome organisation and how simple modulations of them can bring about various chromosomes architectures. Ultimately, all simulations will require experimental validation.

## Funding

This work was supported by the 10.13039/501100001659Deutsche Forschungsgemeinschaft (DFG, German Research Foundation, grant SO 2132/1-1). Costs for open access publishing were partially funded by DFG (grant 491250510).

## Conflict of interest

The authors declare no conflict of interest.

## Glossary

Phase separation: a phenomenon where different molecules in a mixture separate.
Entropy: a property of a system which reflects its disorder. The second law of thermodynamics states that the entropy always increases during an spontaneous process.
Stiffness: a characteristic of an object that defines its rigidity, as opposed to its flexibility.
Reptation: a motion characteristic of polymers that resembles the slithering of snakes entangling each other.
Steric hindrance: a phenomenon of spatial obstruction due to surrounding molecules preventing some conformations to be adopted.
Volume exclusion or depletion attraction: a phenomenon where particles attract each other to decrease the space inaccessible to solvent molecules.
Copolymer: a polymer made of different types of monomers, a heteropolymer.
Osmotic repulsion: a very specific phenomenon where two particles that tend to interact, due for example to depletion attraction, now repeal each other because of steric hindrance between polymers on their surfaces (Fig. 4B).
Crowding: a phenomenon where the increase in the number of surrounding molecules leave almost no room for movement.
